# Oxygen Gas as a Remedy in Disease

**Published:** 1870-05

**Authors:** Andrew H. Smith

**Affiliations:** New York


### OXYGEN GAS AS A REMEDY IN DISEASE.

By ANDREW H. SMITH, M.D., New York.

### CHAPTER I.

History.—The therapeutical history of oxygen dates almost from the moment of the discovery of gas. A few months after he had succeeded in demonstrating oxygen as a separate principle, Priestley discovered its relation to animal life. He found that a mouse, confined in a limited quantity of this gas, lived, at least, twice as long as in a like quantity of common air. This fact led him at once to the suggestion, that this agent might be usefully employed, in cases of disease, in which there was deficient vitality. At the same time, the effect of plunging a burning body into oxygen, inspired him with a misgiving that oxygen could not be inhaled to any considerable extent, without danger of exciting excessive action in the system; that the patient would "*live too fast*"—a phrase which, down to the present day, never fails to rise to the lips of the practitioner, to whom this therapeutic measure is suggested for the first time. Thus early were the possible remedial uses of this agent foreseen, and, at the same time, an erroneous idea advanced, which has maintained its hold upon the professional mind, and prevented much good which might otherwise have been attained.

During the 15 or 20 years following the discovery of Priestley, attention was directed more to the physiological and chemical relations of oxygen than to its use as a remedy. The part played by it in the animal economy was made the subject of investigation by Spallanzani, Fontana, Barthollet, Lavoisier, and others. To Lavoisier belongs the credit of first demonstrating the composition of the atmosphere and the changes produced in the blood by respiration. Following in his footsteps, Spallanzani showed that the consumption of oxygen was in direct ratio to the muscular activity of the animal. For instance, he found that the chrysalis consumed an exceedingly small amount, the caterpillar a much larger proportion, while the active *imago* demanded a very large quantity for its support.

These researches led to the grand discovery, that the new element was the only one, a constant supply of which was necessary for the continuance of life. Food and drink could be withheld for days; even the nitrogen of the atmosphere could be excluded for hours, and yet no serious injury would result. But the animal *began to die* from the instant the supply of oxygen was cut off. No other element stood in this relation to life. No wonder, then, that it was called *vital* air, and that its discovery was thought to have begun a new era for humanity.

The first case in which oxygen was actually employed as a remedy was one reported by Caillens, in 1783. I can find only a reference to this case, which was published in the *Gazette de Sante.* But, in the year following, Jurine, of Geneva, published an essay, in which he cites, at some length, a case of phthisis in a young lady, which was very much benefited by daily inhalations of oxygen. In 1789, Chaptal, of Montpellier, reported two cases of phthisis, in one of which the gas produced great relief, while its use was continued, but in the other the effect was not beneficial.

At about this period the French Government desired an expression of opinion from the academy, upon the value of oxygen as a remedy, and Fourcroy was selected to prepare a report. In this report, and in other works which followed, he resigned himself to a current of speculation, which drifted him far away from the truth. He saw the effect of oxygen in the action of every remedy, even in muriatic acid, the composition of which was not then known. He claims to have employed oxygen in a considerable number of cases of phthisis, and to have noted a rapid improvement for two or three weeks, after which a violent inflammatory action was set up, and the progress of the disease was greatly accelerated.* But his subsequent experiments on animals, in which he describes a state of fever occurring, which eventuated in gangrene of the lungs, led to the suspicion that the gas which he employed contained some irritating impurity, which the imperfect chemistry of the day did not enable him to discover. He became, nevertheless, the founder of a school which interpreted all therapeutical effects by the supposed relation of the agent employed to the oxygen of the system. But it was not until Beddoes began his observations that any valuable practical results were obtained.

In 1789, Beddoes published his book, entitled "Considerations on the Factitious Airs." He was at that time Professor of Chemistry at Oxford, but none the less devoted to the practice of medicine, in which he had already attained a high posi-

*Annales Chimie, 1789.

tion. To him belongs the credit of being the first to approach the subject, without a theory to sustain. It was not until he had accumulated a large number of facts, that he attempted to arrange and classify them. His attempts at generalization were not always attended with the happiest results; but the readiness with which he relinquishes a theory the moment it is found to conflict with fact, gives a rare impression of candor and impartiality to his work. The scope of the work includes observations upon several gases besides oxygen, especially carbonic acid and hydrogen.

His physiological experiments are of great interest. The principal results which he arrived at were the following:—

Oxygen produces a remarkable power of resisting asphyxia. It appears that when the blood contains an unusual amount of oxygen, the animal is better able to support a deficiency of respirable air, or even the presence of an irrespirable gas.

Animals which have respired oxygen resist longer the action of frigorific mixtures.

The action of oxygen seems to be localized principally in the muscular system.

Oxygen is in the highest degree a stimulus to the irritability of the heart and bloodvessels.

The last conclusion is one which succeeding observers will scarcely indorse to the fullest extent. As a stimulant to the circulation, oxygen is certainly far inferior to alcohol; indeed, in many cases, its stimulating effect is scarcely perceptible.

A few isolated cases of success, in the therapeutic use of different gases, encouraged Beddoes to set on foot the project of a Pneumatic Institute, in which this mode of treatment could be tested on an extensive scale. The plan enlisted the co-operation of Sir H. Davy, who gave himself with ardor to the chemical part of the work, and of the eminent engineer James Watt, whose genius left nothing to be desired in the mechanical appliances for administering the gas. Probably a more brilliant triumvirate was never combined in the furtherance of a scientific object.

In pursuance of their plan, a building was erected by public subscriptions. It contained small compartments, the atmosphere of which could be charged with any desired gas. In these rooms, the patients were allowed to pass a certain time daily.

The principal results obtained by the use of oxygen are summed up in the following table, from a review of Beddoes's work, which was published in the *British Library:*

| CASES TREATED. | CURED. | RELIEVED. | NOT BENEFITED. |
|---|---|---|---|
| Obstinate Ulcers | 2 | 2 | -- |
| Leprosy (?) | 5 | -- | -- |
| Spasms | 3 | 2 | -- |
| Gutta Serena | -- | 2 | 3 |
| Chlorosis | 5 | 2 | -- |
| Epilepsy | 1 | -- | 5 |
| Asthma | 10 | 9 | 3 |
| Cancer | -- | 3 | -- |
| Dropsy of the Chest | 2 | 1 | 1 |
| Hypochondria | -- | 1 | -- |
| Dyspepsia | 3 | 1 | -- |
| Dropsy | 2 | 1 | 1 |
| Hydrocephalus | -- | 1 | -- |
| Headache | 2 | 2 | -- |
| Poisoning by Opium | 1 | -- | -- |
| Paralysis | 2 | 1 | 1 |
| Scrofulous Tumors | 2 | 1 | -- |
| Deafness | 1 | -- | -- |
| White Swelling | 1 | -- | -- |
| Scorbutus | 1 | -- | -- |
| Venereal | 1 | -- | -- |
| Melancholy | 1 | 1 | -- |
| General Debility | 1 | -- | -- |
| Continued Fever | 1 | -- | -- |
| Intermittent Fever | 1 | -- | -- |
| Coldness of Extremities | 1 | -- | -- |
| Total | 49 | 30 | 14 |

It will be observed that no cases of phthisis are included in this table. The explanation of this is to be found in the peculiar views entertained by Beddoes, as to the relation of oxygen to this disease. Accepting, without question the reports of Fourcroy, as to the ultimate acceleration of the disease by oxygen, he framed the theory that in phthisis there was a change, either in the constitution of the blood or in the substance of the lung, that favored the absorption of oxygen, which was therefore already present in excess. For this reason he considered oxygen as absolutely contra-indicated.

In scorbutus, on the contrary, he supposed that there was a deficiency of oxygen in the system, which he thought should be supplied by artificial means.

The labors of Beddoes did much towards establishing the true position of oxygen as a therapeutic agent. They demonstrated, on the one hand, that the ideas at first entertained as to its curative power were extravagant; and, on the other, that it was an agent capable of producing good effects, in many cases not reached by ordinary means. The number and variety of diseases, in which the treatment was found beneficial, suggest, at first sight, a certain air of charlatanism, but subsequent observers have corroborated nearly all his statements. Nor, when we consider the physiological relations of oxygen, is it more surprising that its use should be applicable to a large range of cases than that modification of diet should be beneficial in so many diverse diseases.

It is very remarkable that results as satisfactory as those obtained by Beddoes should not have led to a more general adoption of the treatment. But, with the exception of Hill and Thornton, who were contemporaries rather than successors of Beddoes, scarcely any British physician seems to have become interested in the matter, and it was allowed to die out with its original promoters. This was, doubtless, largely due to the difficulties which then beset the production of the gas, and its transportation to the bedside of the patient. Chemical manipulations were then but little understood, and chemical apparatus was very imperfect. Caoutchouc was unknown; and this simple fact alone would have made that very difficult which is now extremely easy. Indeed, when we consider the part which this substance now plays in the manipulation of the gases, it is not too much to say that its introduction was a necessary preliminary to their general use as remedies.

While Beddoes was carrying on his observations in England, the therapeutic use of oxygen was exciting no little attention in Germany. Numerous experiments and observations were made, during the decade preceding the opening of the present century. Prominent among them were those of Girtanner, who, following in the footsteps of Fourcroy, gave arsenic dissolved in nitric acid for a large range of complaints, under the impression that the solution imparted oxygen to the system. He was charmed with the effects of *oxygen*, given in this way, in *intermittent fever.* In the midst of similar speculations and theories, which seem to have taken the place, for the most part, of observations on the practical use of the gas itself, it is not surprising that little real progress was made in determining the true value of the latter.

At Geneva, however, the use of oxygen fell into more practical hands. The results obtained by Jurine served to encourage others. Odier, then a prominent physician at Geneva, took up the new treatment with great zeal, and the *Society for the Advancement of the Arts and Sciences* caused the founding of an institution similar to that of Beddoes. But, as in the former case, this was short-lived; and with its decline the whole subject of the medicinal use of oxygen sank into oblivion. The frightful epidemic of cholera in Europe, in 1832, brought it again into momentary notice, but, as it failed to answer the expectations of those who employed it, it relapsed into its former obscurity.

It is only within the last fifteen years that any serious attempt has been made to bring this agent again into use. Dr. Riadore, it is true, published some observations upon its use, in 1845, recommending it in cases of indigestion, debilitated conditions of the liver and kidneys, nervous affections, asthma, etc. But his cases were not numerous or striking, and failed to arouse the attention of the profession.

In 1857, appeared the first edition of a work on Oxygen, by Dr. S. B. Birch, of London. I have not been able to procure a copy of this edition, and the second, issued in 1868, seems nearly a new work. The writer claims to have presided at the *renaissance* of oxygen, and takes to himself the credit of having instigated all that has been done in the past few years to place its use on a solid foundation. His book consists of a selection of cases, preceded by some general remarks upon the properties and uses of oxygen, its *modus operandi, etc.* His ideas with regard to what he styles the *quasi-nascent* condition of oxygen are very peculiar, and are not borne out by the experience of others. Moreover, his style is singularly obscure, and his book lamentably lacking in practical directions for the administration of the remedy. While constantly insisting upon the necessity of *judiciously* selecting cases for treatment, of *judiciously* administering the gas, and of the *judicious* use of adjuvants, etc., he does not give a single practical rule for determining what is judicious in the premises. The cases which he publishes are very striking, one might almost say marvellous; and the impression which the work, as a whole, is calculated to måke upon the reader is, that in the hands of the author, oxygen is almost a panacea, while, at the same time, it would be hopeless for the general practitioner to attempt to grapple with a treatment so intricate, and demanding such peculiar skill.

In sharp contrast with this work is the article on Oxygen, in Demarquay's *Essai de Pneumatologie Medicale*, published in Paris, in 1866. A little too diffuse, perhaps, it is still plain, simple, and to the point. The author tells his story as of one who has studied the literature of the subject, made some experiments himself, and treated quite a number of cases by the use of oxygen, sometimes successfully, sometimes not. With reference to its use in some diseases, while giving the experience and opinions of others, he states frankly that he has had no experience himself, and does not feel competent to judge of its merits. While it appears to me that some of his experiments on the physiological effects of the gas are defective, yet, others are novel and extremely valuable. His article is marked by perfect candor and frankness throughout, and is by far the best treatise upon the subject extant.

Dr. Hermann Beigel, in his work on Inhalation, published in London, in 1866, presents a few considerations upon the use of oxygen, and cites a number of cases from his practice, in which he has used it with more or less benefit. He invented an apparatus for the production of the gas, according to Fleitmann's process, from the chloride of lime. His treatment of the subject is candid and unpartizan, and his conclusions demand respect.

A new era in the history of oxygen is being inaugurated by the invention of Téssie du Môtay, by means of which the gas can be produced in immense quantities from the atmosphere, and at an insignificant cost. Its possible future, in relation to medicine and hygiene, can as yet be only dimly discerned. When we shall be able to regulate the proportion of oxygen in the atmosphere of the sick-room, as easily as we now regulate the temperature; when closely packed and ill-ventilated tenements can be supplied with this element, the free enjoyment of which is necessary to health; when by its use the contamination of the atmosphere, by the furnaces of factories and machine shops, shall be prevented or counteracted, who can tell what will be the sum-total of the result? Yet all this seems now attainable, whenever the public shall become sufficiently awake to its importance.

### CHAPTER II.

Modes of Preparation and Administration.—In the preparation of oxygen for medical use, *purity* is, of course, of the very first importance. Undoubtedly, many of the effects formerly attributed to oxygen, such as the production of bronchial irritation or inflammation, and even of pneumonitis, were owing to impurities in the gas employed.

The first substance from which oxygen was isolated was the peroxide of mercury, and in all the earlier experiments, the gas employed was obtained from this source. It was not long, however, before cases of ptyalism occurring, warned experimenters of the danger of using the oxides of mercury for this purpose.

Chaptal showed, by carefully conducted experiments, that oxygen so prepared contained an appreciable quantity of the metal. The peroxide of manganese was then substituted, and, finally, chlorate of potash.

Recently, quite a number of processes have been added to the list, so that it now embraces a large range of procedures, by which oxygen may be obtained with more or less facility. I will touch briefly upon the more prominent of these, only one having been found by experience to be really adapted to the use of the physician:—

1. Decomposition of binoxide of manganese. This is accomplished by heating the oxide to a red heat in an iron retort, or by treating it with sulphuric acid. In the first case, a high heat is required, and in the second the acid is disagreeable and dangerous in general practice. Moreover, the gas contains four or five per cent. of nitrogen, from the impurities usually contained in the manganese. If commercial acid is used, it also imparts its impurities to the gas, and among them usually a certain proportion of arsenic. These considerations have led to the complete abandonment of this method in practice.

2. The decomposition of sulphuric acid, or sulphate of zinc. This process depends upon the decomposition of sulphuric acid by heat into oxygen and sulphurous acid, or that of sulphate of zinc into oxygen, sulphurous acid, and oxide of zinc. It is probable that oxygen could be produced in large quantities, in this manner, at a very small cost, so that it would be available for industrial purposes; but, for the use of the physician, the complexity and cost of the apparatus required render it an undesirable method.

3. Process of boussingault. This consists in procuring oxygen from baryta, in utilizing the property which the latter possesses of fixing the oxygen of the air at an elevated temperature, and giving it off again when the temperature is raised still higher. It is difficult to manage, however, and the results are not satisfactory. The apparatus, also, is bulky and expensive.

4. Reaction of sulphuric acid upon bichromate of potash. This reaction results in the production of oxygen and chrome alum. About 16 per cent. of oxygen is yielded by the bichromate. This yield is too small to render the method desirable, aside from the objections belonging to every process, which requires a powerful acid to be placed in unskilled hands.

5. Decomposition of chloride of lime by cobalt. The oxide or any of the salts of cobalt have the property of inducing a species of catalytic action between chlorine and lime, from which free oxygen and chloride of calcium result. An extremely minute quantity of cobalt only is required. If a stream of chlorine gas is passed into warm milk of lime, containing a little of a salt of cobalt, in solution, the chlorine is absorbed, and oxygen is given off; and, at the close of the process, chloride of calcium will have taken the place of the lime. This method of procuring oxygen is known as Fleitmann's process. Now, by using chloride of lime, we have the chlorine and the lime united in one substance; and, by merely adding the cobalt, and pouring on a little hot water, the process is greatly simplified. The gas, however, contains considerable chlorine, and the yield is small in proportion to the quantity of material employed. This process is the one recommended by Dr. Beigel, for prepairing oxygen for medical use; and his recommendation is sustained by Birch, who, however, prefers compressed oxygen, when it can be obtained. I have given the method a trial, but, in my hands, the quantity of gas was so small, and the quality so inferior, that I abandoned its use. However, as the cost is very slight, it might be used with advantage in office practice, where a large stationary apparatus could be employed, and where arrangements could be made for washing the gas through an alkaline solution to remove the chlorine. But, for use at the bedside, an apparatus, small enough to be portable, would not yield the gas in sufficient quantities.

It remains to consider the method of obtaining oxygen by the decomposition of chlorate of potash. This substance having the formula $KO, ClO^5 = KCl + O^6$, is broken up by heat into oxygen and chloride of potassium.

By mixing with the chlorate a little peroxide of manganese, the disengagement of the oxygen proceeds with greater rapidity and requires much less heat. It is usual to invoke the action of catalysis to explain this, but it seems to me to be owing simply to the facility with which the manganese conducts the heat and diffuses it through the whole mass. Chlorate of potash alone is an exceedingly bad conductor of heat, as is also chloride of potassium. Hence the action of slight degrees of heat is confined to that part only of the mass exposed, which is in contact with the retort. But the manganese, being a heavy metallic substance, transmits the heat readily from particle to particle of the salt. Any other substance having an equal conducting power will do as well, provided it will not combine with oxygen. I have succeeded admirably with the black oxide of copper. Sand may be used, but with less advantage, as it is comparatively a poor conductor. It is generally stated that this process yields perfectly pure oxygen gas.

This, however, is not the case if the evolution is at all rapid, as the gas will then be slightly contaminated with chlorine. There is also another impurity, not noticed in any work on chemistry which I have seen. Pure oxygen, as is well known, is invisible, yet the product from chlorate of potash has usually more or less of a smoky appearance, when first evolved. If the gas be allowed to stand for half an hour, or an hour, it will lose this appearance, while the glass vessel in which it is contained will be seen to have a deposit on its inner surface. Under the microscope this deposit is found to consist of minute crystalline particles. If enough of these be collected to respond to chemical tests, they will be found to be chlorate of potash. It would seem, then, that a small portion of the chlorate, instead of being decomposed by the heat, is sublimed unchanged; and such is its insolubility that it is not separated from the gas, except by repeated washings. Not the least harm results, however, from inhaling it, as I have demonstrated repeatedly in my own person.

The quantity of gas procured by this process is very great, amounting in round numbers to 500 cubic inches, for each ounce of the chlorate of potash, or 39 per cent. by weight. The quantity yielded renders this method peculiarly adapted for use in the sick-room, where portability of apparatus and material is much to be desired. Until recently, I have employed a copper flask in which to heat the materials. But I found inconvenience to result from this form of container, inasmuch as the entire quantity of the chlorate was heated at once, resulting in a tumultuous and often unmanageable evolution of the gas. To obviate this difficulty, I have had constructed the apparatus figured in the annexed cut. It consists essentially of a brass retort, in the form of a cylinder, nine inches long and one and a-quarter inches in diameter, resembling in shape a very large test-tube. To the open extremity of this retort is fitted a cover of cast iron, held in place by a clamp, which catches upon a projecting flange surrounding the mouth of the retort. This clamp is tightened by means of a screw. Passing through the cover is the tube carrying the gas into the wash-bottle, and which is arranged at a right angle to the retort. The latter is, therefore, in a horizontal position, and is supported by its connection with the wash-bottle, which in its turn is firmly fastened to a board, forming the base of the whole apparatus. The tube before referred to passes to the bottom of the wash-bottle, and has, near its lower extremity, a great number of very small holes, through which the gas escapes in fine bubbles. This is important, as it insures a much more perfect washing of the gas. Another tube, merely passing through the cap of the wash-bottle, provides for the passage of the gas into the bag, from which it is inhaled.

The retort is but half filled with the mixture of chlorate of potash and peroxide of manganese, and this quantity is distributed along its whole length, to within an inch of the cover, thus leaving nearly one-half of the diameter of the retort free for the passage of the gas. The heat of a Bunsen burner or of a powerful spirit-lamp is employed, beginning first at the closed extremity of the retort, and moving it along as the material becomes exhausted. The wash-bottle is half filled with a solution of caustic potash.

The advantage of this apparatus is, that only a small portion of the material is heated at a time, and the rapidity of evolution is under perfect control. By having two retorts, and using them alternately, a continuous supply may be kept up, sufficient for any emergency. The whole apparatus, including the bag, may be easily packed in a box ten inches square, and five inches deep, and a supply of gas may be generated in 15 minutes, at the house of the patient.

In using black oxide of manganese in connection with chlorate of potash, it is important that it should be free from protoxide, and from any combustible substance. Neglect of this precaution may lead to an explosion.

The process of Téssie du Môtay is as follows:—Manganate of soda is exposed to a very high heat in iron retorts, and while in that condition, a current of atmospheric air is passed over it. This results in the absorption by the salt, of a large portion of the oxygen which the air contains. The current of air is then shut off, and a current of superheated steam substituted. The steam withdraws from the manganate of soda the added quantity of oxygen, and carries it with it to a condenser, from which the oxygen passes in a pure state into the gasometer. The salt is then subjected to the action of a second portion of air, followed again by a curreut of steam, and thus the process goes on indefinitely. The manganate of soda retains its activity, and loses nothing in weight, so that the only consumption is that of fuel.

For use in the sick-room, the gas may be compressed into cylinders of copper or iron, and thus rendered conveniently portable.

In localities sufficiently near to a factory, this is destined to supersede all other methods of supplying oxygen for medical purposes. The gas is perfectly pure, and the quantity which can be compressed into even a small cylinder is sufficient to meet the requirements of any case likely to occur.

The method of administration of oxygen is very simple. The gas, being in a bag or in a gasometer, is conveyed to the mouth or nostrils of the patient, by means of a flexible tube, terminating in a mouth-piece of glass, hard rubber, or ivory. This being taken into the mouth, or held to one nostril, the patient breathes the oxygen, mingled with a greater or less proportion of common air, one or both nostrils being free for the admission of the latter. The proportion of gas is regulated by the size of the orifice through which it escapes. During *expiration*, the rubber tube is compressed between the thumb and index-finger. When the patient is not able to do this for himself, it may be done by an attendant, who, by watching the movements of the chest, soon catches their rhythm. I prefer this plan to the use of an inhaler, with a complicated system of valves, which always offers an impediment to respiration.

The quantity given will vary from one to two gallons daily, which is sufficient, in some chronic cases, to 80 or 100 gallons, which may be required in urgent dyspnœa. In chronic cases it should be given from a very small orifice, so that the inhalation of four or five gallons will occupy 15 to 30 minutes. Feeble patients should take it in the recumbent position.

The inhalations may be repeated morning and evening, or less frequently, as the case may demand. Some very striking results have followed, when the interval was as great as three days. On the other hand, when respiration is very much obstructed, it may be necessary to give the gas almost constantly, and but little diluted.

Knowing the capacity of the bag employed, and bearing in mind that an adult usually respires from eight to ten pints of air per minute, it is easy to judge approximately of the quantity of oxygen which is being inhaled.

The plan of surcharging the atmosphere of a room with oxygen, and allowing the patient to remain in it for a certain period, has this disadvantage, that, to retain the oxygen, ventilation must be sacrificed. If the room be so large as to do away with this objection, the quantity of oxygen required will be greater than can generally be supplied. These considerations have led to the abandonment of this mode of administration.

Dr. Birch lays great stress upon the gas being given in what he calls a "*quasi-nascent*" condition; that is, he thinks it should be inhaled at once as rapidly as it is generated, or, if not, that it should be kept under pressure until wanted for use. It is enough to say that he brings forward no facts to sustain the alleged advantage of this plan, and that, moreover, others who have not followed it have obtained equally good results.

### CHAPTER III.

Physiological Action of Oxygen.—In regard to the physiological action of oxygen, the first question to be determined is, whether it is possible to cause the blood to take up more oxygen than it receives from the atmospheric air; whether the point of saturation is not attained in ordinary respiration. On this point, there was formerly but one opinion. It was thought that there was practically no limit to the power of the blood to absorb oxygen. This idea was no doubt in part based upon the known energy of combustion in pure oxygen gas, and the supposed identity of that process with the retrograde metamorphosis of animal tissue. It was held that the inhalation of pure oxygen would induce rapid chemical action within the body, that a state of general inflammation would ensue, and that destructive metamorphosis would be so much more active than the process of reconstruction, that the vital machinery would soon be spoiled and rendered incapable of continuing its action.

But, after a time, it was observed that these extreme results did not actually take place, that an animal could remain for a number of hours in pure oxygen, without sustaining any apparent injury. This led certain observers to the conclusion that the blood-corpuscle became saturated with oxygen, when common air was breathed, and that it would take up no more, no matter how much was presented to it in the air-cells of the lungs. This view was defended by Regnault and Reiset, who endeavored to sustain it by the following experiment:—They confined animals in oxygen, and after a time examined the gas, and found that it contained no more carbonic acid than would have been exhaled in the same time if the animals had respired atmospheric air. That these experiments were not conclusive, will become apparent as we proceed.

On the other hand, was cited the fact that animals die in a period varying from three to eighteen hours, if confined in an atmosphere of oxygen, and that the tissues present an unusually florid aspect, approaching to a vermilion hue. These observations I believe to be no more conclusive than the others. On this point, Demarquay says: "Science had already taught what our experiments have confirmed, that animals can live without danger in an atmosphere of pure oxygen, and for a much longer time than in the same volume of air. But beyond a certain limit, these animals at last succumb, and one may then satisfy himself that the medium in which they have respired is still capable of relighting an ignited body; a very evident proof that death has taken place from the oxygen itself, and not from any alteration which it may have undergone from admixture with the carbolic acid exhaled." *

As the test referred to above, that of relighting an extinguished taper, the extremity of the wick being still red hot, is constantly relied upon to prove the respirability of the gas after such experiments, it is well to state at the outset that it is entirely worthless. This is shown by the following experiment:

EXPERIMENT I.—Two parts of pure oxygen were mixed with one part of carbonic acid, prepared by the action of sulphuric acid upon marble. A pint jar was filled with the mixture, which sufficed to relight a taper four times.

Demarquay himself states that ten per cent. of carbonic acid, mixed with oxygen, is sufficient to render the latter incapable of sustaining life; yet we find, by this experiment, that the test which he relies upon would indicate its respirability, when containing 33 per cent.

The apparatus employed by Demarquay in his experiments, which are essentially similar to those of his predecessors, consisted of a large cylinder, furnished with two openings, through one of which the animal was introduced, while to the other a tube was fitted, connected with a reservoir of oxygen. The animal, having been placed in the cylinder, a large quantity of oxygen was introduced by the tube, the amount being sufficient to drive out the air in the apparatus, which escaped by the other opening. When it was judged that the cylinder was filled with pure or nearly pure oxygen, both apertures were closed. The animals experimented upon were common fowls, pigeons, and rabbits.

The result of these experiments was, that when the animals were allowed to remain in the apparatus for the space of an hour and three-quarters, and were then killed, nearly all the tissues of the body were found reddened to a greater or less extent, but the venous blood retained its darker hue. Two

* Essai de Pneumatologie Médicale, p. 644.

rabbits were allowed to remain until death took place, which, in one instance, was at the end of 14 hours, and in the other, after 17 hours. At the close of each experiment the gas was found to relight a taper.

These experiments coincide in their results with one of my own, in which the conditions were similar:

Experiment II.—*June* 10, 1860.—A rat was confined in a jar containing about a gallon of pure oxygen, and inverted over water. At the end of two and a-half hours death took place. On opening the body, all the internal organs were found to be of a bright-red color.

It will be observed that, in both these instances, no provision was made for removing the carbonic acid and other products of respiration, and that the gas must have become excessively impure. In the following experiments this omission was corrected:

Experiments III., IV., and V.—*July* 15, 1869 —A pigeon and two mice were confined respectively three, four, and five hours in jars of oxygen, having a strong solution of caustic potash in the bottom, under a stage upon which the animal rested. The jar was so arranged, at the same time, that a small stream of oxygen, from a rubber bag, was constantly flowing into it, and escaping by an aperture of like size. The solution of potash absorbed the carbonic acid, while the gradual change of the atmosphere within the jar was sufficient to prevent a sensible accumulation of other impurities.

*These animals, when killed, did not present any appreciable change in the appearance of the tissues.*

Experiment VI.—*August* 25, 1869.—At 3 P.M., a pigeon was placed in a jar of oxygen, of the capacity of 300 cubic inches, and the jar inverted over a solution of potash. The following morning the animal was found dead. Upward of 150 cubic inches of oxygen had been converted into carbonic acid, and absorbed by the potash, the liquid rising in proportion in the jar. On opening the body, *no unusual redness of the tissues was observable.* The feathers of the bird, and also the sides of the jar, were wet with the condensed moisture of the breath. The animal was also in a constrained and uncomfortable position, which, doubtless, hastened its death.

The conclusion which I draw from these experiments is, that the lively red color of the lungs, heart, liver, etc., which are described, and which I have myself seen, does not depend upon hyperoxygenation alone, but also upon a coincident retention of carbonic acid in the tissues. The color pervades the intervascular substance as perfectly as the natural coloring-matter pervades the muscular fibre. It cannot, therefore, be ascribed to simple increase of vascularity.

The following experiments show, that in Demarquay's observations, the oxygen is as little chargeable with the death of the animals as with the change in the color of their tissues:—

Experiment VII.—*August* 13, 1869.—A mouse was confined in a jar of oxygen inverted over a solution of caustic potash. At the end of 25½ hours, during which he had neither food nor drink, he was dull and stupid, but, when released, ate greedily, and was soon as lively as ever.

Experiment VIII.—*August* 16, 1869.—A tin box, seven by ten inches, and six inches deep, open at the bottom, and having the top of glass, was placed in a shallow vessel containing a solution of potash. A little above the surface of the solution was arranged a false bottom of wire-cloth, which formed the floor of the apparatus. A circular opening on one side of the box was fitted with a projecting rim, soldered to its edge. To this rim or collar a cap was fitted, and the joint was made air-tight by an india-rubber band stretched around it. This opening was for the purpose of introducing the animal to be experimented upon. A small tube passing into the box was connected with a large reservoir of oxygen. By means of a small stop-cock, the amount of gas passing into the apparatus was so regulated, that a bubble would escape every second or two from under the edge of the box. Within the box was placed an open vessel containing chloride of calcium, to absorb the moisture from the breath, and another vessel with dilute nitric acid, to take up the ammonia exhaled. Food, water, and a quantity of tow for a bed having been provided, a mouse was introduced into the apparatus, and the aperture closed air-tight. Oxygen was then admitted freely, for some time, until all the air was expelled, when the stop-cock was closed to the point already indicated. The animal ate, drank, and arranged his bed, and acted in every particular as mice generally do, until the third day, when he buried himself in the tow, and seemed very quiet. By this time his excretions gave to the gas, which escaped from the apparatus, a very sickening smell.

At the end of four days, the mouse was removed and transferred to a cage, where he recovered at once his accustomed liveliness, and appeared no worse for his unique experience.

This single experiment is sufficient to overthrow the theory of hyperoxygenation of the blood, as the term is generally understood, and to show that the fatal results heretofore observed, as well as the peculiar *post mortem* appearances, were the result of the admixture of the products of respiration with the gas inhaled.

Are we, then, to accept the conclusion of Regnault and Reiset, that inhaling pure oxygen makes no difference with respiration? Clinical observation and facts derived from experiments teach us clearly to the contrary.

The quantity of oxygen in the blood under normal conditions is extremely variable. This follows from the varying exigences of the system. The transition from perfect repose to active exertion implies increased molecular action and increased consumption of oxygen. The blood-corpuscles are the carriers of oxygen, and, as their number remains the same, each one must assume a greater burden. To explain all the phenomena resulting from the inhalation of oxygen, it is not necessary to assume the absorption of more than corresponds with the extreme limit of this healthy respiratory demand. All the analogies of Nature lead us to suppose that this limit coincides with the point of complete saturation of the blood. To assume a margin beyond it is to suppose a provision against an emergency which can never arise. It is contrary to the economy of Nature that the blood should have the capacity for absorbing more oxygen than Nature can supply.

My view is then, that if pure oxygen be taken into the lungs, only as much will be absorbed by the blood as would be taken up from the air under circumstances involving the greatest possible physiological demand for oxygen. I know that it has been asserted that blood agitated in a vessel with oxygen will assume a livelier red than when agitated with common air. This, however, is a mistake. The change will take place more promptly with oxygen, but the hue will be in the end the same. We may, therefore, assume, that if the blood and the air be brought into sufficiently intimate contact in the lungs, the corpuscles will become saturated with oxygen from the ordinary atmosphere.

Experiment IX.—*August* 20, 1869.—A quantity of defibrinated sheep's blood was divided into two portions. One portion was thoroughly agitated with oxygen, and quickly assumed a bright-red hue. The other was agitated in the same way with common air. The change took place more gradually, but, eventually, when the two jars were placed side by side, no difference in the color could be distinguished.

The portion agitated with air was then placed in a vessel filled with oxygen, which was closed tightly, while its interior was made to communicate with a delicate manometer. After the lapse of an hour, during which the vessel was frequently agitated, no change had taken place in the height of the fluid, in the instrument, thus indicating that no additional oxygen had been absorbed.

How, then, is this appearance of superoxygenation of the blood to be accounted for, since it never occurs when atmospheric air is respired? The conditions which obtain while breathing oxygen, without removing the products of respiration, are entirely *sui generis.* They differ from that observed when air is substituted, in that the portion of carbonic acid may become much greater without destroying life. They differ, also, from the effect produced by confining an animal in a mixture of carbonic acid and oxygen, since, in the latter case, the change is abrupt, while in the former it is very gradual. The experiments of Count Morrozo, and of Bernard, show what an immense difference, in the effect upon the animal, results from this circumstance. I offer the suggestion, therefore, that the red stain of the tissues is the result of the prolonged action of carbonic acid retained in the blood—life, meanwhile, being kept up, and the activity of retrograde metamorphosis sustained by a *maximum absorption of oxygen.*

When a considerable quantity of pure oxygen is inhaled, there is usually a sensation of freedom about the chest, as if respiration were easier. Some persons describe a feeling of warmth beneath the sternum, such as results from inhaling a slightly-stimulating vapor. Sometimes a slight degree of vertigo is produced. Generally there is a tendency of the blood to the surface, and the hands and feet, if previously cold, become warm. In some cases, this change of the circulation is accompanied by a prickling sensation. The pulse is sometimes accelerated, but more frequently remains unchanged. In cases of debility, it is often reduced in frequency. The temperature is but little changed, if at all. I have sometimes observed a disposition to yawn constantly during the inhalation, and there is generally an inclination afterward to sleep. All these effects are more marked when the gas is inhaled fasting.

In reference to the effect of the inhalation of oxygen upon the amount of carbonic acid formed, and of urea excreted, there has been as yet but little research. The experiments of Regnault and Reiset, upon the first point, have been already referred to. The subject, however, is beset with difficulties, and much caution is required in accepting the results of experiments as to the amount of carbonic acid exaled, when breathing a greater or less proportion of oxygen. Different results will be obtained at different times, when breathing the same medium, under apparently the same conditions as to diet, stage of digestion, exercise, etc. The slightest bodily exertion, or even mental excitement, will vitiate the experiment. In experiments with animals, eructations of gas from the stomach will sometimes add largely to the per centage of carbonic acid obtained.

My experiments, on this point, have brought out an (to me) unexpected result, *viz.*, that the inhalation of a considerable quantity of oxygen is followed, within a few moments, by a temporary *decrease* in the amount of carbonic acid exhaled, as is shown in the following table:—The experiments were made upon myself.

| | Hour. | Cubic inches of oxygen inhaled. | $CO_2$ exhaled per minute. |
|---|---|---|---|
| EXPERIMENT XI., July 29, 1869. | 4.35 P.M. | | 21 c. in. |
| | 4.45 " | 600 | |
| | 5 " | | 19 |
| | 5.18 " | | 20 |
| EXPERIMENT XII., August 3. | 3.25 " | | 22 |
| | 4 " | 400 | |
| | 4.10 " | | 16 |
| EXPERIMENT XIII., August 5. | 12.15 " | | 19 |
| | 12.25 " | 700 | |
| | 12.30 " | | 16½ |
| | 12.45 " | | 18 |
| EXPERIMENT XIV., August 5. | 2 " | | 19½ |
| | 2.50 " | 400 | |
| | 2.55 " | | 19½ |
| EXPERIMENT XV., August 5. | 2.55 " | | 19½ |
| | 3.15 " | 1200 | |
| | 3.35 " | | 17½ |
| EXPERIMENT XVI., August 12. | 6 A.M. (fasting) | | 18 |
| | 6.15 " | 500 | |
| | 6.40 " | | 17 |
| | 7 " | | 17½. |

EXPERIMENT XVII.—*August* 4, 1869.—A pigeon was placed in a jar containing 300 cubic inches of oxygen, and the jar inverted over a solution of caustic potash. After 20 minutes, the oxygen was removed and replaced by common air. In 30 minutes the volume of air had decreased 13 cubic inches. The following day, the same pigeon was confined again, in the same quantity of air, for the same period, not having previously inhaled oxygen. The decrease amounted to 18 cubic inches. There was no evidence that the health of the animal had been injured by the previous experiment.

The manner in which oxygen produces this effect is not easily explained. It is possible that its immediate action may be like that of alcohol, which is known to cause a diminution of the carbolic acid exhaled from the lungs. This is the more probable from the similarity of its other effects, when well marked, to those which alcohol produces.

Notwithstanding this temporary decrease of carbonic acid, I am of the opinion that the ultimate effect of oxygen is to cause its increase. The increase is probably small, and it would, doubtless, be extremely difficult to demonstrate it conclusively, under normal conditions of activity. Still, it seems to me, that the result of the following experiments could hardly be attributed to mere accident:—

EXPERIMENT XVIII.—*August* 12 *to* 24, 1869.—Three observations were taken daily of the amount of carbonic acid exhaled by myself. In all, 20 observations were made, in seven days, nearly every hour of the day being represented.

The average of these observations gave 17.2 cubic inches per minute. During the four following days, eleven similar observations were made—the conditions remaining the same, except that from six to ten gallons of oxygen were inhaled each day in divided doses. The average of these gave 18.9 cubic inches, as the amount of carbonic acid exhaled per minute.

These results, while they coincide with the generally received opinion as to increased activity in the retrograde metamorphosis, as resulting from the use of oxygen, show, nevertheless, that the increase is confined within narrow limits, and thus confirm the view already expressed, that saturation of the blood-corpuscles with oxygen is quickly attained, is a *strictly physiological condition*, and in no way necessitates the setting up of any morbid action within the system.

To test the effect of oxygen upon the amount of *urea* excreted, I made the following experiment:—

Experiment XIX.—*December* 8 *to* 22, 1869.—The urine for each 24 hours was carefully preserved, and the amount of urea estimated according to Haughton's second formula. The result is given in the following table:

| Date. | Gallons of Oxygen Inhaled. | Urea in Grains. |
|---|---|---|
| December 8 | -- | 641 |
| " 9 | 8 | 624 |
| " 10 | 8 | 561 |
| " 11 | 10 | 472 |
| " 12 | 6 | 472 |
| " 13 | 6 | 590 |
| " 14 | 12 | 550 |
| " 15 | 14 | 552 |
| " 16 | 18 | 510 |
| " 17 | -- | 542 |
| " 18 | -- | 601 |
| " 19 | -- | 546 |
| " 20 | -- | 556 |
| " 21 | -- | 495 |

This table shows a rapid decrease in the amount of urea, during the first four days after beginning the inhalations of oxygen. It then increased again, but did not attain to the former figure. With the cessation of the oxygen, there is again an increase. The average of the days without oxygen is 563 grains; of those with oxygen, 541 grains.

So far as these experiments go, they indicate that oxygen causes a *decrease* in the amount of urea formed. This is surprising, if we are to consider urea as the result of *oxidation* of tissue, as is generally held. More extended observation is required, before it would be warrantable to call in question the views so ably enunciated upon this point; but, should it be established, that the continued use of oxygen really diminishes the excretion of urea, it would place the latter substance in analogy with the smoke resulting from combustion, a product, it is true, of the combustion, but, at the same time, a measure of the incompleteness of the process.

The quantity of uric acid in the urine is rapidly diminished by the daily use of oxygen. This fact, suspected by Dr. Golden (*Lancet*, March 10, 1866), has been fully established by Kollmann, of Munich.

In the course of the experiments described above, I observed a very striking diminution of the coloring matter of the urine. At the commencement of the experiment, the urine was very high-colored, but, within 24 hours, it became very pale, and remained so for several days after the oxygen was discontinued. This paleness was not owing to an excess of water, as the specific gravity never fell below 1.022, and was usually above 1.025.—*New York Medical Journal.*

